# Protective role of butyrate in obesity and diabetes: New insights

**DOI:** 10.3389/fnut.2022.1067647

**Published:** 2022-11-24

**Authors:** Arianna Mayorga-Ramos, Carlos Barba-Ostria, Daniel Simancas-Racines, Linda P. Guamán

**Affiliations:** ^1^Facultad de Ciencias de la Salud Eugenio Espejo, Centro de Investigación Biomédica (CENBIO), Universidad UTE, Quito, Ecuador; ^2^Escuela de Medicina, Colegio de Ciencias de la Salud Quito, Universidad San Francisco de Quito USFQ, Quito, Ecuador; ^3^Facultad de Ciencias de la Salud Eugenio Espejo, Centro de Investigación en Salud Pública y Epidemiología Clínica (CISPEC), Universidad UTE, Quito, Ecuador

**Keywords:** butyrate, obesity, fiber, short-chain fatty acid (SCFA), diabetes

## Abstract

Studies in human microbiota dysbiosis have shown that short-chain fatty acids (SCFAs) like propionate, acetate, and particularly butyrate, positively affect energy homeostasis, behavior, and inflammation. This positive effect can be demonstrated in the reduction of butyrate-producing bacteria observed in the gut microbiota of individuals with type 2 diabetes (T2DM) and other energy-associated metabolic alterations. Butyrate is the major end product of dietary fiber bacterial fermentation in the large intestine and serves as the primary energy source for colonocytes. In addition, it plays a key role in reducing glycemia and improving body weight control and insulin sensitivity. The major mechanisms involved in butyrate regulation include key signaling pathways such as AMPK, p38, HDAC inhibition, and cAMP production/signaling. Treatment strategies using butyrate aim to increase its intestine levels, bioavailability, and improvement in delivery either through direct supplementation or by increasing dietary fiber in the diet, which ultimately generates a higher production of butyrate in the gut. In the final part of this review, we present a summary of the most relevant studies currently being carried out in humans.

## Introduction

The World Health Organization (WHO) defines obesity as an abnormal fat accumulation that may impair health (1). The evidence of the relationship between the gut microbiome and the development of obesity and type 2 diabetes mellitus (T2DM) has been rising for the last decade (2). The gut microbiota plays a pivotal role in regulating energy homeostasis. Among other host factors, this balance between energy intake and expenditure relies on the microorganisms and their metabolites, helping in nutrient processing, nutrient access regulation, and storage in the body by secreting hormones and mediators of energy homeostasis (3). Some glycemic alterations that cause a detrimental cascade effect have also been linked to chronic inflammation, cardiovascular diseases, and gut dysbiosis, where the loss of several butyrate-producing bacteria has been observed (4).

The short-chain fatty acids (SCFAs) are ingested through diet or produced by fermentation of non-digestible fiber by gut microbiota; the three major SCFAs produced are butyrate, propionate, and acetate (5). After production or consumption, butyrate, the SCFA with the most important systemic effects, is absorbed rapidly in the gut and acts as a source of energy and a signaling molecule in numerous cell types (6). It has been reported to have metabolic effects on obesity and glucose homeostasis. In a recent study, individuals with obesity and T2DM showed a decreased abundance of butyrate-producing bacteria and downregulation of genes related to butyrate production (7). Despite these findings, its exact role remains unclear since obese individuals present higher fecal butyrate concentrations than the control group, with similar diet consumption (8, 9). Restoration of butyrate-producing bacteria and butyrate levels by ingesting butyrate-rich foods or dietary fibers that lead to butyrate production might provide new treatment options for T2DM and obesity-related metabolic diseases (5). Here we review the basic mechanisms that explain the role of butyrate in this context.

## Sources of butyrate in food

SCFAs, including butyrate, are present in high amounts in milk and milk derivatives from different mammals. Bovine fat milk and its derivatives are a great source of butyric acid, e.g., butter (∼3 g/100 g), goat’s cheese (∼1–1.8 g/100 g), parmesan (∼1.5 g/100 g), whole cow’s milk (∼0.1 g/100 g) (10). Human milk (HM) has also been reported as a source of butyrate, where the concentration measured in HM samples from healthy women fluctuates between 0.15 and 1.93 mM in colostrum and 0.16–1.97 mM in mature milk. Considering a median butyrate concentration of 0.75 mM in mature HM, a breastfed infant could receive a daily dose of butyrate of approximately 30 mg/kg body weight (11).

Salatrims, a fat calorie replacer commonly used in the food industry, is also a source of dietary butyrate. Salatrim has triglyceride mixtures in which butyric acid is inter-esterified with a long-chain fatty acid moiety such as stearic acid (12, 13). Since butyrate is esterified at the α(sn-3) position (14, 15), pancreatic lipase can cleave triacylglycerols releasing free fatty acids (FFA) in the small intestine (16, 17). To prevent digestion -and absorption- in the upper part of the gastrointestinal tract, butyrate might be esterified to a dietary fiber such as butyrylated or tributyrin, in which butyrate is esterified to triglycerides; as a result, these esterificated form of butyrate have also been shown to increase colonic butyrate concentrations (18, 19).

In most human clinical and rodent studies focused on obesity and diabetes, butyrate is supplied orally as sodium butyrate, which has an unpalatable flavor and odor. Novel strategies have been developed recently to improve palatability and the release and absorption of butyrate in the digestive tract (20). Coating sodium butyrate with cellulose-based capsules has been one approach to delay the release in the intestinal tract (21). According to pharmacological and clinical data from the literature, butyric acid is considered a safe drug. Therapeutic doses (150–300 mg) have shown no clinical side effects (22). Even when higher doses up to 2,000 mg/day, no adverse reactions have been observed (22, 23)

On the other hand, dietary fiber is used by some bacteria in the gut microbiota for butyrate production in two different ways: (I) Direct, where fiber acts as a substrate for bacterial fermentation producing butyrate, (II) Indirect since bifidogenic fibers help to increase the abundance of bifidobacteria, which increase butyrate production indirectly (24).

Maybe, the most efficient way to influence the composition of intestinal and colon microbiota is by ingestion of dietary fibers such as inulin-type fructans, xylooligosaccharides, arabinoxylans, arabinoxylan oligosaccharides, β-glucans, and oligofructose (25–28). Important foods to increase intestinal butyrate are complex polysaccharides not easily digested by saliva and pancreatic amylases. For instance, resistant starch, a group of molecules that resist digestion, may be added or fortified into bread and cereals (29) but is also found naturally occurring in some legumes, cooked potatoes, and unripened bananas. Studies have shown that resistant starch potentiates butyrate production and yields more butyrate than non-starch polysaccharides (30, 31). Other non-easily digested polysaccharides producing butyrate include cereal breakfasts, such as barley and oats, containing β-glucans, which are also naturally present in edible mushrooms and seaweed (32). Finally, inulin, on the other hand, is mainly found in artichokes, onion, and chicory roots (33) are also good foods to increase intestinal butyrate.

A high-fiber diet can cause gastrointestinal discomforts, such as gas, bloating, and constipation in patients with Crohn’s disease, irritable bowel syndrome, or ulcerative colitis. This is why a gradual increase in fiber intake is recommended for everyone (34).

## Biosynthesis and butyrate absorption

Although butter is the most abundant source of dietary butyrate (up to 3 g per 100 g), the best way to increase the amount of intestinal butyrate is by consuming non-digestible carbohydrates (complex polysaccharides) to increase *in situ* production by human gut microbiota (35). Intestinal butyrate is produced by obligate anaerobic bacteria through fermentation (36). Most human butyrate producers belong to the *Firmicutes* phylum including species such as *Clostridium butyricum, Clostridium kluyveri, Faecalibacterium prausnitzii, Butyrivibrio fibrisolvens, Eubacterium limosum* (37–39).

Although several routes for the production of butyrate have been described, in human gut microbiota, butyrate is mainly synthesized from acetyl-coenzyme A (Ac-CoA) obtained from the breakdown of complex carbohydrates (e.g., xylan, starch) as a precursor (18, 37). Subsequently, two molecules of AcCoA condense into acetoacetyl CoA, and after several consecutive steps, it is transformed into butyrate (40). The final step in butyrogenesis is the conversion of butyryl-phosphate into butyrate by butyrate kinase encoded by the *buk* gene or butyryl-CoA to butyrate by butyryl-CoA: acetate-CoA transferase, encoded by the *but* gene (41). In addition to the colonization of the colon by butyrogenic bacteria, it has been proposed that cross-feeding interactions between *Bifidobacterial* strains and *F. prausnitzii* may ultimately enhance butyrate production (42).

Whether butyrate is ingested through the diet or produced locally in the intestine from dietary fiber, it is absorbed into the enterocytes by diffusion and delivered through the portal vein into the liver and systemic circulation (43, 44). Due to its size and hydrophobicity, butyrate, like propionate and acetate, are absorbed through a non-ionic diffusion across the apical membrane of colonocytes (45, 46). Sodium-coupled monocarboxylate transporter 1 (SCMT1) utilizes colonic concentration of Na^+^ to internalize SCFAs within colonocytes. It has been described that SCMT1 transports propionate acetate at a slower rate compared to butyrate transport. The solute carrier family 5 member 8 (SLC5A8) is considered the primary butyrate transporter across the apical membrane of the colonocytes (47, 48).

## Butyrate and liver metabolism

The liver is the master organ for regulating energy homeostasis, particularly for lipid and glucose metabolism regulation. The liver plays a central role in the development of obesity-associated metabolic alterations. It is, therefore, highly relevant to outline the effect of butyrate on regulating lipid metabolism and liver function. Recent studies showed that butyrate supplementation reduced serum triglyceride levels and the respiratory exchange ratio in high-fat diet (HFD)-fed animals compared to controls with HFD only, suggesting that butyrate may exert its effect by promoting fatty acid oxidation (49–53). In addition, butyrate also reduced lipid content in brown adipose tissue (BAT) and, to a lesser extent, in the liver and muscle (51). Additionally, demonstrating its protective role, butyrate supplementation in animals with HFD resulted in a significant reduction of proinflammatory serum markers (TNF-α, MCP-1, and IL-1β) compared to the markers of animals with HFD only (50).

Although butyrate mechanisms of action are unclear, previous studies show that butyrate modulates the AMP/ATP ratio activating the AMPK pathway to promote oxidative metabolism (decrease lipid synthesis and increase lipid oxidation) in the liver and adipose tissue (53). In addition, butyrate increases the percentage of oxidative type fibers (actively using lipid oxidation for ATP biosynthesis) in skeletal muscle by activating AMPK and p38 (54) and increases mitochondrial function in skeletal muscle (49) and liver (50).

## Butyrate as a regulator of body weight

In addition to its well-known effects on intestine function (55), butyrate is a critical link between gut microbiota and the regulation of host energy homeostasis. Results from several groups have provided evidence supporting this role. For instance, chronic sodium butyrate supplementation in food (49, 51, 53, 54, 56) or orally delivered *via* gavage (50, 57–59) reduces body weight gain and fat mass of mice fed with a HFD compared to mice fed with an HFD alone, suggesting that butyrate can prevent or contribute for the treatment of diet-induced obesity (DIO). Also in agreement with this regulating role, intraperitoneal (IP) injection of sodium butyrate for ten consecutive weeks reduced body weight gain of rats treated with butyrate (60). A mechanism explaining the effect of butyrate on reducing body weight and fat mass is the activation of lipid oxidation initiated by butyrate in BAT and the liver (51). In addition to the increased energy expenditure and lipid oxidation, reduced food intake, also described as an effect of oral butyrate supplementation but not an intravenous injection, may contribute to decreased body weight gain induced by butyrate and reduced fat mass (51). The lack of effect of intravenous injection of butyrate suggests a role of this SCFA in regulating the gut-brain circuit.

Although there is evidence that sodium butyrate supplementation in standard diet-fed rats reduced weight gain (55), the effects of butyrate under chow diet-fed conditions remain controversial. There are contradicting results from other studies claimed that a chow diet plus 1% or 5% butyrate did not significantly affect body weight in mice (50, 61) and even that offspring rats of mothers fed a 1% butyrate in a chow diet had higher body weight (62). Major milestones of butyrate action and results from key therapeutic trials are summarized in Table 1.

**TABLE 1:** Therapeutic interventions with butyrate supplementation.

Population	Type (concentration)	Design approach	Trial details	Metabolic effects	Study
**Obesity and metabolic syndrome**					
– Children with obesity (*n* = 48)	Sodium butyrate (20 mg/kg body-weight per day)	Randomized controlled trial	Duration: 6 months administration: oral doses: 1 per day (no schedule specified)	– Decrease in waist circumference and BMI – Significant reduction of fasting insulin, HOMA-index, and LDL – Significant downregulation of peripheral miR-221 expression and a significant decrease of fasting serum ghrelin	(90)
– Healthy lean males (*n* = 9) -males with obesity (*n* = 10)	Sodium butyrate (4 g per day)	Clinical trial	Duration: 4 weeks administration: oral doses: 2 per day (no schedule specified)	– Males with obesity exhibit decreased oxLDL-induced trained immunity. – No effect observed in the counts of neutrophils, lymphocytes, or monocytes	(91)
– Patients with metabolic syndrome (*n* = 24)	Sodium butyrate (4 g per day) + autologous fecal transplantation	Randomized clinical trial	Duration: 4 weeks administration: oral doses: 1 per day (no schedule specified)	– Decrease in HbA1c, total cholesterol and triglycerides – No effects in BMI, hepatic and peripheral insulin sensitivity, fasting insulin and fasting glucose	(87)
– Healthy lean males (*n* = 9) -males with metabolic syndrome (*n* = 10)	Sodium butyrate (4 g per day)	Clinical trial	Duration: 4 weeks administration: oral doses: 2 per day (no schedule specified)	– Healthy lean males exhibit improvements in peripheral and hepatic insulin sensitivity – No effect observed in individuals with metabolic syndrome	(89)
**Diabetes**					
– Patients with T2DM (*n* = 42)	Sodium butyrate (3.6 g per day)	Randomized controlled trial	Duration: 6 weeks administration: oral doses: 6 per day (before and after 3 main meals)	– Decrease in systolic and diastolic blood pressure – Slight decrease in blood sugar 2-h postprandial but no statistically significant difference with placebo group	(85)
– Patients with T1DM (*n* = 53)	Sodium butyrate (3.6 g per day)	Randomized controlled trial	Duration: 12 weeks administration: oral doses: 2 per day (no schedule specified)	– No effects in inflammatory markers, kidney parameters, HbA1c, metabolites or gastrointestinal symptoms	(92)
– Patients with T2DM (*n* = 60)	Sodium butyrate (600 mg per day) + inulin (10 g per day)	Randomized controlled trial	Duration: 45 days administration: oral doses: 2 per day (no schedule specified)	(2017) – Decrease in diastolic blood pressure – Decrease in fasting blood sugar and hip-to-waist ratio during combined (sodium butyrate + inulin) administration (2020) – Downregulation of genes: TLR2/4, NF-κB1, Caspase-1, NLRP3, IL-1β and IL-18 (related to pyroptosis cell death)	(86, 93)
– Patients with T1DM (*n* = 30)	Sodium butyrate (4 g per day)	Crossover randomized controlled trial	Duration: 4 weeks administration: oral doses: 2 per day (no schedule specified)	– No effects in BMI, energy intake, fasting glucose or total insulin dose – No effects in β-cells autoimmunity or innate immunity regulation	(88)

BMI, body mass index; HbA1c, glycated hemoglobin; HOMA-Index, homeostatic model assessment for insulin resistance; IL-1β, Interleukin-1β; IL-18, Interleukin-18; NF-κB1, Nuclear factor κB1; NLRP3, NOD-, LRR- and pyrin domain-containing protein 3; oxLDL, oxidized low density lipoprotein; TLR2/4, Toll-like receptors 2/4; T1DM, Type 1 diabetes mellitus; T2DM, Type 2 diabetes mellitus.

## Butyrate and diabetes

**FIGURE 1:**
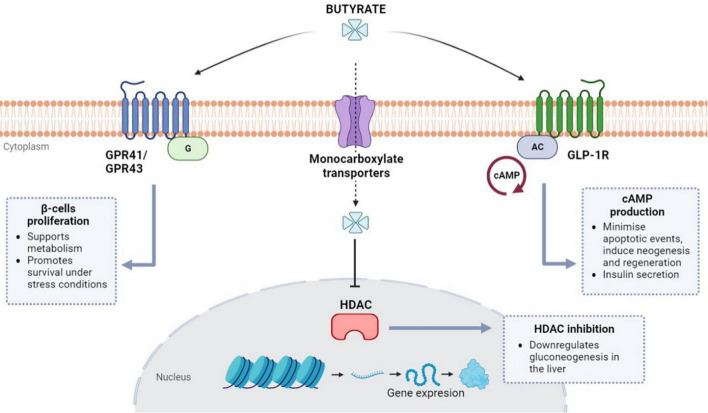
Butyrate regulation pathways in β-cells (pancreatic islets). Butyrate is involved in β-cells metabolism *via* G protein-coupled receptors (GPRs) GPR41 and GPR43. Butyrate can also enter the cells via monocarboxylate transporters and inhibit histone deacetylases (HDAC) which are thought to be involved in gluconeogenesis signaling. Butyrate can also regulate the cAMP signaling for insulin secretion *via* glucagon-like peptide-1 receptors (GLP-1R).

As it has been extensively documented in the literature, insulin resistance can be attributed to a decrease in receptor sensitivity together with the functional impairment of β-cells in the pancreatic islets (63). Histological studies of human islet tissue have shown that butyrate has a protective effect against oxidative and mitochondrial stress promoting the survival of β-cells *in vitro* (64, 65). Remarkably, initial analysis of the conditions associated with β-cells autoimmunity in children at risk of type-1 diabetes mellitus (T1DM) found a reduction in the average population of butyrate-producing bacteria (66, 67). There is evidence that butyrate is involved in the metabolism of β-cells in the pancreatic islets due to its interaction with G-protein coupled receptors (GPR) like free fatty acid receptors FFAR3 (GPR41) and FFAR2 (GPR43), as seen in Figure 1 (64, 68, 69). In light of this association, one study suggested that butyrate could be responsible for a proliferative effect during *in vitro* mouse intestinal organoid development due to its interactions with GPR41 and 43 receptors (70).

Animal data in obese mice have shown that butyrate administration rapidly decreased fasting insulin levels (50, 71). As a result, aside from its protective role in β-cells, butyrate has been proposed as a direct regulator of insulin secretion *via* GPR-mediated signaling. However, this direct role remains unconfirmed and controversial (72). Nevertheless, recent findings suggest sodium-butyrate treatment can indirectly enhance insulin secretion by repressing β-cell key functional genes in rat islets (73). More evidence about the indirect role of butyrate during insulin secretion has emerged after studies reported its involvement with glucagon-like peptide-1 (GLP-1) secretion from intestinal L-cells (74, 75). Activation of the GLP-1R (receptor) genes, also present in β-cell, and subsequent GLP-1 release can also be induced by butyrate (76). GLP-1 has the potential to minimize apoptotic events and induce neogenesis and regeneration of β-cells *via* cAMP (cyclic adenosine monophosphate) upregulation (77). Production of cAMP can also elicit postprandial-like insulin secretion by accelerating the glucose-dependent closure of ATP-regulated potassium channels (77–79).

On the other hand, the inhibitory activity of butyrate toward histone deacetylases (HDAC) has also been extensively described in literature due to HDAC involvement in transcriptional regulation, metabolism, metastasis, oncogenesis, and ischemic brain events (80, 81). HDACs have also been linked to hyperglycemia by promoting gluconeogenesis in the liver; therefore, becoming an important target to regulate glucose levels *via* the administration of HDAC inhibitors like metformin (a first-line antihyperglycemic drug for T2DM treatment) (82, 83). Type-2 diabetic animal studies have revealed that butyrate has similar effects to metformin in reducing insulin resistance and other T2DM-associated conditions (60). In addition, the role of butyrate-mediated HDAC inhibition has been described as an enhancer in the differentiation and maturation of β-cells in neonatal porcine islets (84). Altogether, there is strong evidence about the crucial role of butyrate and its interactions with insulin-secreting β-cells. The potential regulation of gluconeogenesis *via* HDAC and the robust induction of insulin secretion *via* GLP-1 appoint butyrate administration as a potential target for research in diabetes treatment.

Consequently, several therapeutic interventions have already been conducted to assess the effects of butyrate supplementation in diabetic patients and patients with obesity and metabolic syndrome (Table 1). Some trials reported a positive outcome after treatment with oral butyrate with a reduction in the patient’s blood pressure and blood sugar levels (85, 86). In addition, other trials also found significant reductions in HbA1c (hemoglobin-A1c: glucose linked to hemoglobin in red blood cells), total cholesterol, and triglycerides (87). On the other hand, several studies contradict some of these findings and report no significant effects on sugar levels, insulin sensitivity, and secretion (87, 88). One study suggests that butyrate therapy benefits healthy individuals, but this outcome is not reflected in patients with metabolic syndrome (89). Overall, there are still some limitations on these data, which may prevent direct comparisons and conclusions. Some of these trials may be conditioned by the short duration of butyrate administration, small sample size, lack of placebo control, and variability within the target population. Moreover, butyrate combination therapies with other potential antidiabetic drugs are still unexplored, so further research is encouraged.

## Concluding remarks

In addition to the described effects on intestinal function and metabolic control, the system-level impacts of butyrate remain elusive, and the detailed molecular mechanisms responsible for butyrate action in host and microbial cells are still a very active research field. On the other hand, to detail the effects of butyrate on host metabolism and further promote butyrate as a therapeutic approach for designing novel clinical trials, it is critical to identify its effects on different animals and humans and evaluate different doses, treatment times and delivery methods. In addition, although many studies support the role of butyrate as an essential mediator in host metabolic control, some of its effects remain controversial.

Highly relevant questions in this exciting field are still awaiting elucidation. They should be decoded, including but not limited to determining the best conditions and food sources for butyrate production by gut microbiota *in situ*, absorption of dietary and microbially produced butyrate under different physiological and pathological conditions, the regulatory mechanisms of butyrate at a cellular and systemic levels and the potential of using it as a therapeutic alternative in some obesity-associated metabolic alterations.

## Author contributions

CB-O and LG: conceptualization and research/investigation. AM-R, DS-R, and CB-O: writing—original draft preparation. LG and DS-R: writing—review and editing. LG: supervision. All authors contributed to the article and approved the submitted version.
